# Transcultural adaptation and validation of the Spanish language version of the questionnaire OQLQ for the assessment of quality of life in orthognathic patients

**DOI:** 10.4317/jced.55366

**Published:** 2018-12-01

**Authors:** Rocío Sánchez-Burgos, Carlos Martínez-Gimeno, Ignacio Arribas-García, Guillermo Gómez-Oliveira, Modesto Álvarez-Florez, Alberto García-Hernández, Ricardo Martínez-Martínez

**Affiliations:** 1 Head of Department of Oral and Maxillofacial Surgery

## Abstract

**Background:**

Orthognathic surgery is the cornerstone of the treatment of dentofacial deformities, which have a great psychological and social impact on the life of the patient. Patient satisfaction and the impact on quality of life have recently become clinical parameters of growing importance. The aim of this study was to undertake a transcultural adaptation, translation to Spanish and validation of this version of the questionnaire OQLQ, used to measure quality of life in the context of Spanish culture.

**Material and Methods:**

Validation of the OQLQ questionnaire to the Spanish language was carried out through the methodology of translation and back translation, conceptual equivalence and piloting. The Spanish version was applied through a cross-sectional study to a total of 50 patients undergoing orthognathic surgery.

**Results:**

The adapted and validated version showed adequate metric properties of reliability, change sensitivity and validity. In this study, a positive impact of orthognathic surgery on the specific quality of life was evident in 96% of patients, with an average improvement of 58% with respect to the initial score.

**Conclusions:**

Dentofacial deformities have a marked negative impact on the lives of patients, with orthognathic surgery being a therapeutic tool of great value in improving the quality of life in social, functional and aesthetic terms. The pilot test of this Spanish language version of the OQLQ proved valid for the assessment of quality of life in Spanish-speaking orthognathic patients or those with a Spanish culture.

** Key words:**Orthognathic surgery, quality of life, validation studies, dentofacial deformities, patient satisfaction, treatment outcome.

## Introduction

Scientific evidence exists showing that dentofacial deformities have a very negative impact on the quality of life of affected persons, and that orthognathic surgery is a therapy with a success rate of 85% (1-9). This benefit depends not only on achieving the objectives set by the medical team, but also on many other more subjective factors, such as the patient expectations, associated anxiety levels or the secondary psychological stress generated by the condition.

With effect from the 1990s researchers started to examine the impact of surgery on the psychological and social spheres of the patient, with the initial publications highlighting its beneficial effect (2,10-12). This resulted in psychological factors becoming important for the success of the treatment and being recommended to be included in a greater patient-centred diagnostic process (5,13). A systematic review of the literature published in 2013 included 21 studies on the quality of life in orthognathic surgery, though only three studies undertook measurements over time (14). Accordingly, very little exact information is available about the longitudinal changes in quality of life arising during the treatment process. Nor is much known about the long-term duration of these positive effects, though they seem to remain for at least two years after surgery (15,16). Thus longer term studies are required.

Many non-specific questionnaires exist for use in oral and maxillofacial surgery. The most used in orthognathic surgery are the OHIP (Oral Health Impact Profile), the SF36 (Short Form Health Survey) (17), and the OHSQ (Oral Health Status Questionnaire). In 2000 Cunningham et al developed the first questionnaire designed specifically to measure the quality of life in patients with dentofacial deformities, which was validated with favourable results concerning internal consistency and reliability (18,19). This tool, called the Orthognatic Quality of Life Questionnaire (OQLQ), has now become the most used to evaluate quality of life in patients who undergo orthognathic surgery (14,17,20-23). It consists of 22 questions divided into four domains that address facial aesthetics, oral function, concern about the deformity and social aspects. The individual responses can vary from N/A (does not apply to you) to 4 (it bothers me a lot), giving a total score ranging from 0 to 88. Higher scores indicate a worse quality of life and a greater perceived need for treatment. The questionnaire was drawn up using various sources of data to obtain the different questions, including a literature review and interviews with physicians and patients. Its validity was checked using the SF-36 and the visual analogue scale as comparative models. It has been used to assess both the quality of life of patients with dentofacial deformities and the impact of surgical treatment, showing greater sensitivity than the usual questionnaires (18,20,22,24,25). It has proven to be the tool with greatest validity for the evaluation of patients with dentofacial deformities (21,22,24,26). It has been used in over 30 studies dealing with various aspects of quality of life in orthognathic surgery, and is now the most used in this field. At present it has been translated and validated into German (20), Serbian (27), Portuguese (Brasil) (28,29) (29), Arabic (30) and Chinese (25).

## Material and Methods

**Figure 1:**
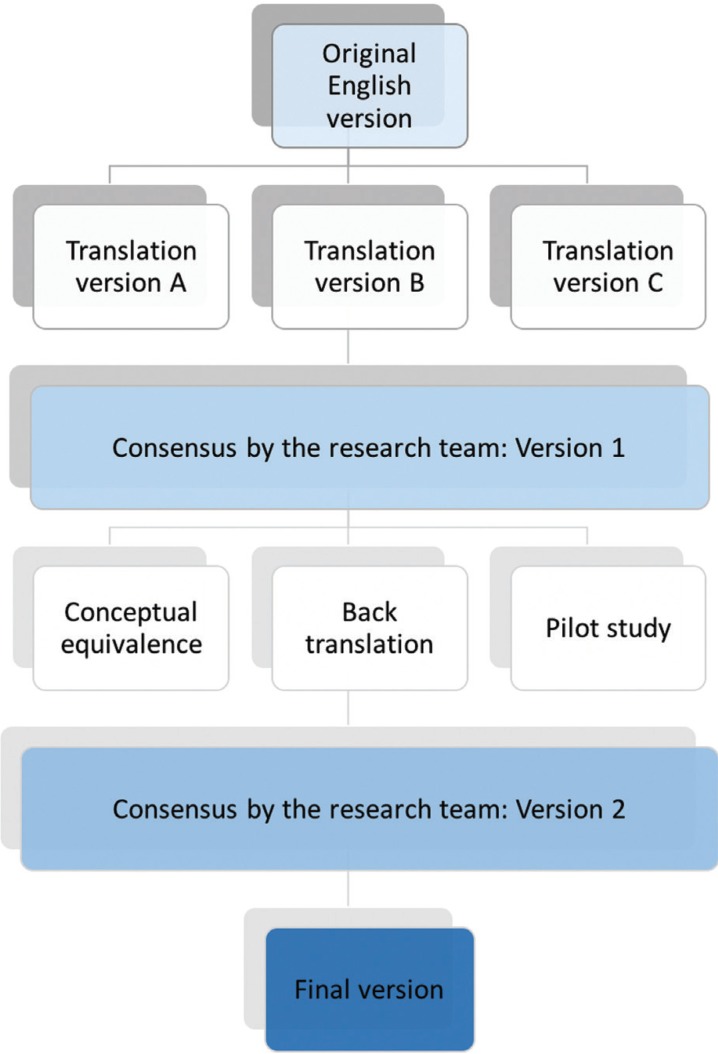
Schematic process of the transcultural adaptation of the OQLQ questionnaire to Spanish.

The aim of this study was to obtain a Spanish version of the OQLQ for orthognathic surgery using a validated process of transcultural adaptation. The validation of the questionnaire to Castilian Spanish was done first by translation and back-translation, and then submitting the translated version to a process of conceptual equivalence and applying this version in a pilot study (Fig. 1). First, it was necessary to obtain the explicit permission of the authors of the original questionnaire to undertake this project.

**Figure 2:**
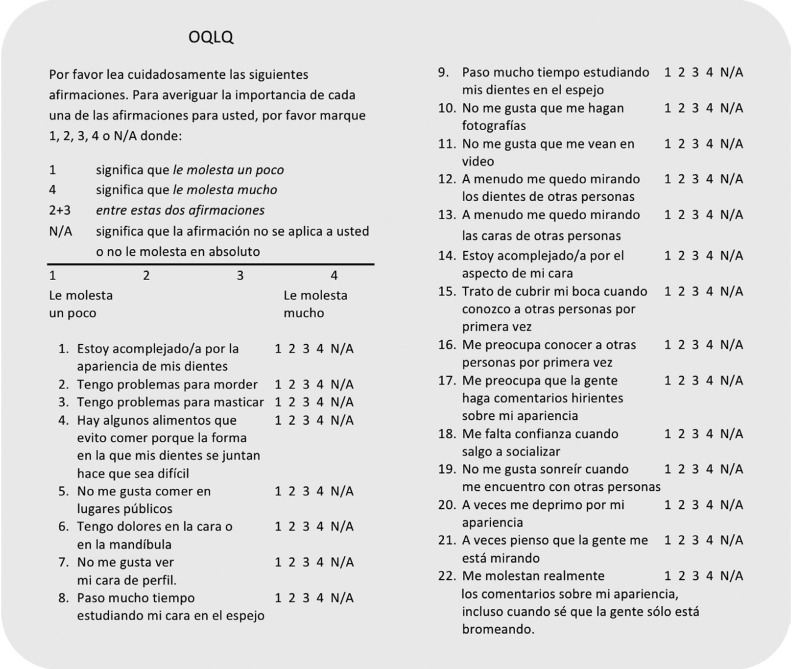
Spanish version of the OQLQ questionnaire.

The first step in the process of transcultural adaptation consists of the translation of the original version from English to Spanish by three bilingual professionals whose mother tongue was Spanish, producing the three translated versions A, B and C. The research team then discussed the work and agreed on a Version 1 of the questionnaire. This version was then submitted to a process of conceptual equivalence involving both the research team and English language experts. It was then back translated to English by a professional translator whose mother tongue was English and a comparison made between the original English version and this back-translated version. Finally, the Spanish version was given to pilot sample to verify its metric properties, (Fig. 2).

This piloting was done with a retrospective, observational cross-sectional study. It included 50 random patients treated by the Oral and Maxillofacial Surgery service of the University Hospital of the Canary Isles (HUC). The patients were obtained from the database of the service according to the following inclusion criteria: patients with bone maturity who had a dentofacial deformity treated by orthognathic surgery between January 2007 and December 2016. Patients were excluded if they had prior or posterior surgery altering the facial aesthetics. Patients were also excluded if they hemifacial microsomia, cleft lip and palate or other craniofacial malformations, or if they had undergone distraction osteogenesis. The patient was contacted by telephone and requested to complete the questionnaire for two points in time: their situation before treatment and their current postoperative situation. They could receive the questionnaire by e-mail or attend the hospital in person.

For the electronic version the patient gave their e-mail address to the researcher and was then sent both the information sheet and the informed consent form together with the questionnaire. Marking acceptance was a necessary condition for the study. All the information, both sent and received, is anonymous and associated with an alphanumeric code and stored in an encrypted database. For the version completed in person the patient was duly informed about the survey and gave informed consent prior to completing the questionnaire. Anonymity was also guaranteed.

The information was processed in a specially designed database. The statistical study, depuration and recoding of the database was all undertaken with IBM SPSS Statistics 24.0. The statistical process of validation was done by study of the viability, reliability, sensitivity and validity. The reliability was evaluated by analysis of the internal consistency using Cronbach’s alpha. The sensitivity to change was evaluated by use of the effect size index and bivariate tests of statistical significance (Wilcoxon). The construct validity was evaluated by doing a factorial analysis to measure the concordance of the dimensions with the original model.

At all times care was taken to safeguard the confidentiality of the data with sufficient guarantee and in accordance with the principles of the Declaration of Helsinki. The study was approved by the Ethics Committee of the University Hospital of the Canary Isles.

## Results

The preliminary version obtained after the process of transcultural adaptation was used in a pilot study to determine its metric properties of reliability, sensitivity and validity. We initially consulted 98 patients selected randomly from the Oral and Maxillofacial Surgery service database. Of these, 50 (51,02%) agreed to participate. The overall mean score on the questionnaire regarding the situation before treatment was 45.92 (± 24.64) and the median was 49. The maximum score was 86 and the minimum 0. The mean score after treatment was 15.46 (± 13.88) and the median was 13. The maximum was 70 and the minimum 0.

Of the 50 patients, 48 (96%) reported a better score after treatment. The mean percentage improvement in the overall score after surgery (average after – average before/average before * 100) was 58%.

**Figure 3:**
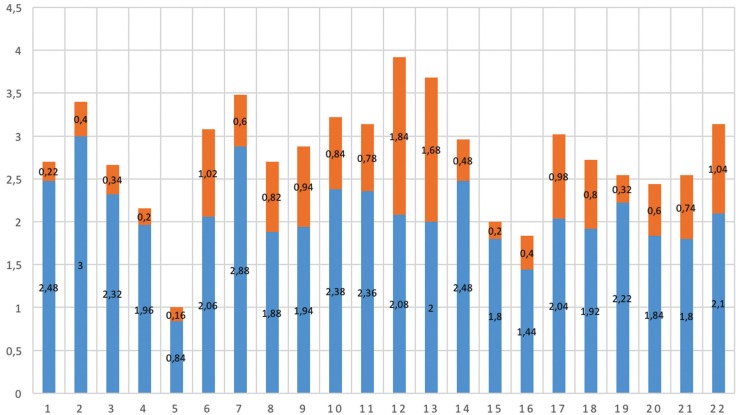
Stacked columns showing the distribution of the mean scores for each of the 22 questions before (blue) and after (orange) treatment.

Individual analysis according to each question showed an improvement in the mean score for all answers after treatment, being statistically significant in 20 of the 22 questions (Fig. 3). The mean score before treatment was 2.08 whereas after treatment it was 0.7. Analysis of the domain of facial aesthetics showed that the mean scores for all the questions fell significantly after treatment (*p* < .001). Questions number 1 (“I am self-conscious about the appearance of my teeth”) and number 7 (“I don’t like seeing a side view of my face”) showed the greatest reduction in the mean score from before to after the treatment. In the domains of oral function and social aspects all the mean scores also improved significantly after surgery. The question showing the greatest difference between the means was number 2 (“I have problems biting”). Finally, in the part about concern about deformity, all the questions showed an improvement in the mean scores, though this was not significant for questions number 12 (“I often stare at other people’s teeth”) and number 13 (“I often stare at other people’s faces”) (*p*=0.11 and *p*=0.06, respectively).

The reliability was assessed via the internal consistency calculating Cronbach’s alpha. The four domains of the questionnaire were analysed, measuring the internal consistency according to the homogeneity of the responses within each domain. In all cases the Cronbach’s alpha was greater than 0.70. The highest Cronbach’s alpha value was obtained for the questions in the domain exploring social aspects (0.958) and the lowest value (though still valid) occurred in the domain analysing oral function (0.776). The sensitivity was examined with the sensitivity to change concept (responsiveness) by calculating the effect size. The greatest effect size was obtained in the domain relating to facial aesthetics (1.21) and the lowest in that concerning social aspects, 0.74. An analysis of the sensitivity to change was done using the Wilcoxon signed rank test, comparing the mean range of the responses before and after treatment. The difference was statistically significant for all the questions (*p*<0.001), except for questions 12 (“I often stare at other people’s teeth”) and 13 (“I often stare at other people’s faces”) ( Table 1).

**Table 1:**
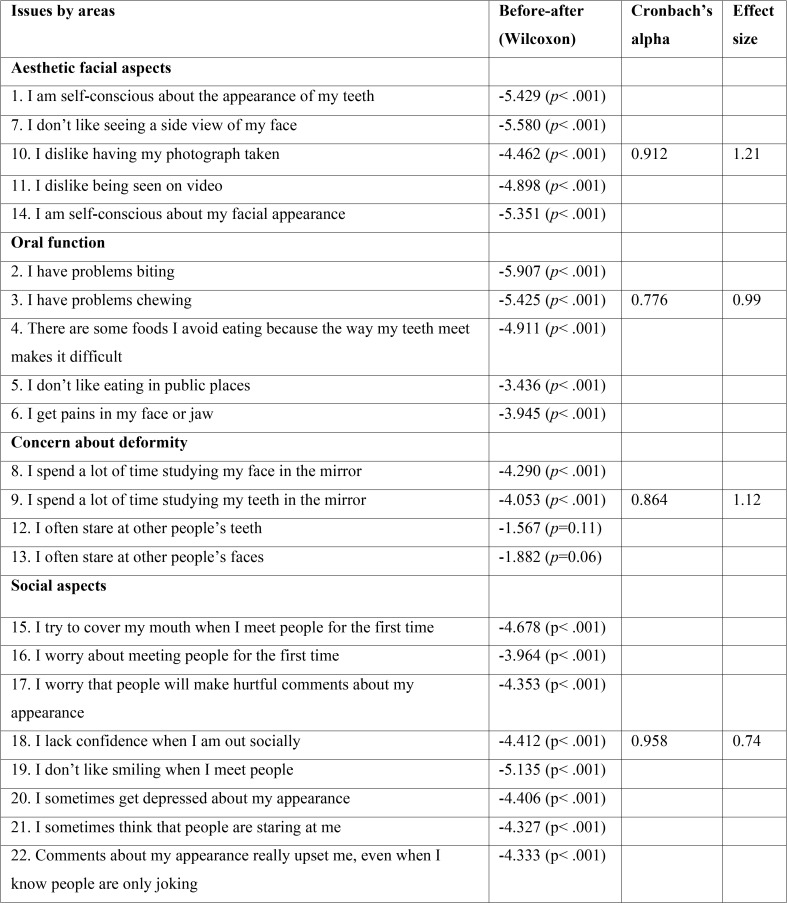
Values of the Wilcoxon signed-rank test, Cronbach’s alpha and effect size for the different questions and domains on the questionnaire.

The content validity was evaluated by applying factorial analysis to group the responses and study the grouping according to the underlying factors, then making a comparative analysis compared with the original version. Using the criterion for factor extraction that considers the principal components to have values greater than one, we identified five factors that explained 79% of the variance. The domain on the original questionnaire that explores social aspects of the deformity includes the subgroup of questions 15 to 22 ( Table 2, in red), with a very high factorial load and homogenous within component 1, as well as the questions exploring facial aesthetics ( Table 2, in blue). Notable for component 2 was the factorial load for questions 8, 9, 12 and 13 ( Table 2, in green), which comprise the questions relating to oral function. Notable for component 3 was the factorial load for questions 2 to 6, the subgroup exploring the last domain of the original test, concern about the facial deformity ( Table 2, in yellow).

**Table 2:**
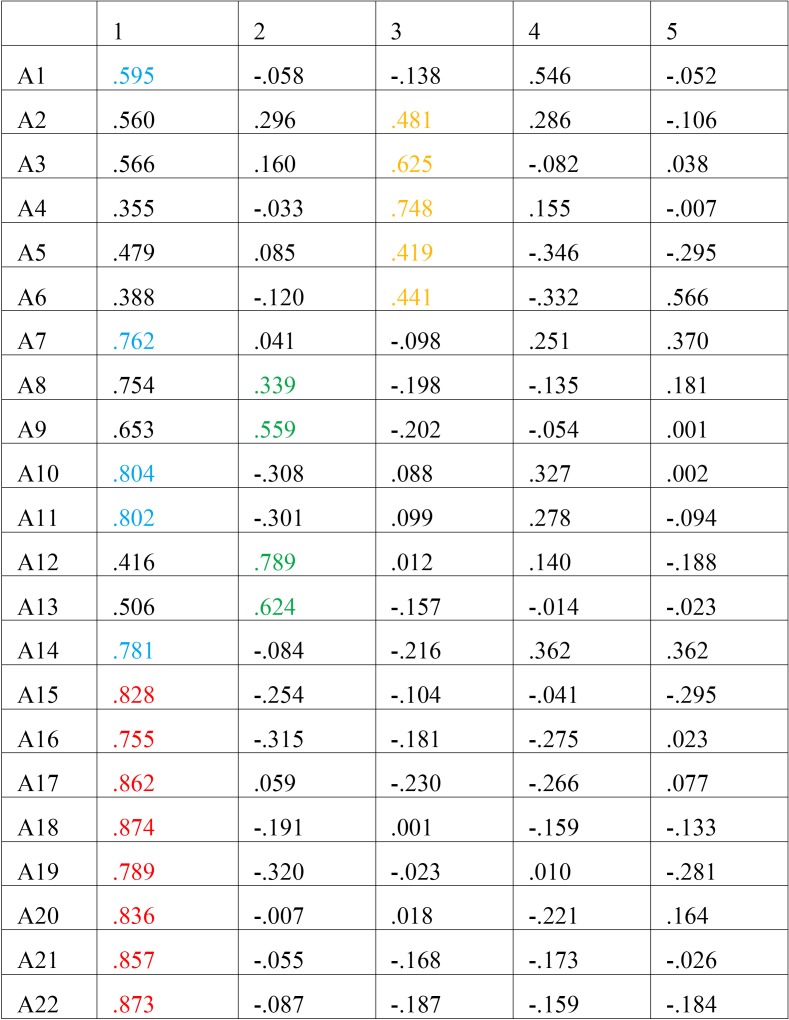
Component matrix.

After jointly examining all the preliminary statistical validation parameters and their metric properties we considered the preliminary version to be valid as the definitive version of the process of transcultural adaptation.

## Discussion

The use of questionnaires in medicine has increased greatly over recent years as a result of new views on the diagnostic and therapeutic processes, which are now patient centred. Indeed, questionnaires now constitute a basic tool in the decision-making process. Given the cultural plurality of the medical literature, the use of validated and correctly adapted questionnaires is essential in order to be able to extrapolate results between populations and convert a study in a valid international tool. Before a questionnaire can be applied in a different population and cultural context to that where it was originally developed it must undergo a process of transcultural adaptation. The aim of this is to produce an instrument that equates to the original, since a simple literal translation may exclude certain cultural nuances. In the present study we followed the recommendations of the International Test Commission for the validation of questionnaires, thereby guaranteeing an adequate methodology.

The most used method of semantic and cultural adaptation is that of translation back-translation by bilingual experts, followed by a conceptual analysis of the resulting version, which is then piloted to measure the psychometric properties.

Reliability is the ability of an instrument to produce similar results each time it is applied. This reliability is assessed through its internal consistency, which evaluates the degree of homogeneity in the responses to the same concept. In our analysis using Cronbach’s alpha we obtained values above 0.7 for all the domains, demonstrating that the new version has adequate internal consistency.

Sensitivity is based on the quality of an instrument to detect true cases. This concept was examined using the effect size, which represents the difference between the values before and after treatment. As a general rule the effect size is considered to be small if it is less than 0.2, from small to moderate if it is from 0.2 to 0.5, moderate to large if it is from 0.51 to 0.79, and large if it is above 0.79. In our study the highest value was obtained for facial aspects, with an effect size of 1.21, followed by concern about the deformity and function, both of which had high values.

Sensitivity to change was analysed using the Wilcoxon signed-rank test to compare two related measurements, in our case the responses before and after treatment. The difference was statistically significant for all the questions (*p*<0.001), except for questions number 12 (“I often stare at other people’s teeth”) (*p*=0.11) and 13 (“I often stare at other people’s faces”) (*p*=0.06), in the domain concern about the deformity. In both these latter cases the initial scores were similar to the mean before treatment (2.08), but after treatment both scores (1.84 and 1.68) considerably surpassed the overall mean (0.7). Nevertheless, the other two questions in this domain, numbers 8 (“I spend a lot of time studying my face in the mirror”) and 9 (“I spend a lot of time studying my teeth in the mirror”) did show significant differences after treatment. Thus, in this pilot study the questions relating to concern about the deformity centred on physical self-examination benefitted significantly from the surgery, whereas those focused on the observation of physical characteristics in other persons resulted in a non-significant benefit. The comparative analysis with the original version of the factorial matrix in our study showed a very similar structure, with correspondence between the domains, thereby showing adequate construct validity.

Accordingly, the process of validation and posterior piloting of our Spanish language translation of the OQLQ showed adequate reliability, sensitivity and validity. The results of this pilot study demonstrate that this Spanish version of the OQLQ is suitable for evaluating both the impact of the dentofacial deformity on the quality of life of the patient and the effect of orthognathic surgery. It also highlights, yet again, the great benefits of orthognathic surgery in the life of affected patients.
